# Supplementary material for: XBP1-mediated transcriptional regulation of SLC5A1 in human epithelial cells in disease conditions
Source: Res Sq. 2023 Jul 21:rs.3.rs-3112506. Preprint. [Version 1] doi: 10.21203/rs.3.rs-3112506/v1 (PMC10371076; doi:10.21203/rs.3.rs-3112506/v1)
Supplement: 1 [file NIHPPRS3112506V1-supplement-1.pdf]

**Supplementary Figure 1.** XBP1 upregulates SGLT-1 expression in CFPAC-1-dF cells.

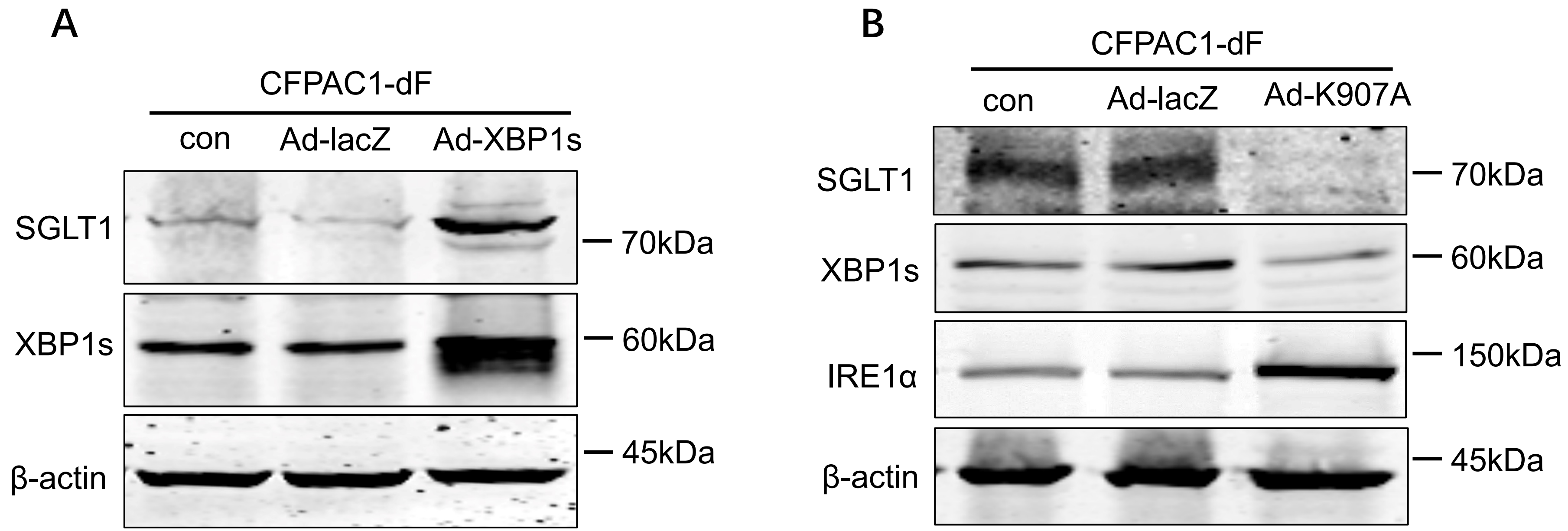

**Supplementary Figure 2.** XBP1 upregulates SGLT-1 expression in HK-2 cells.

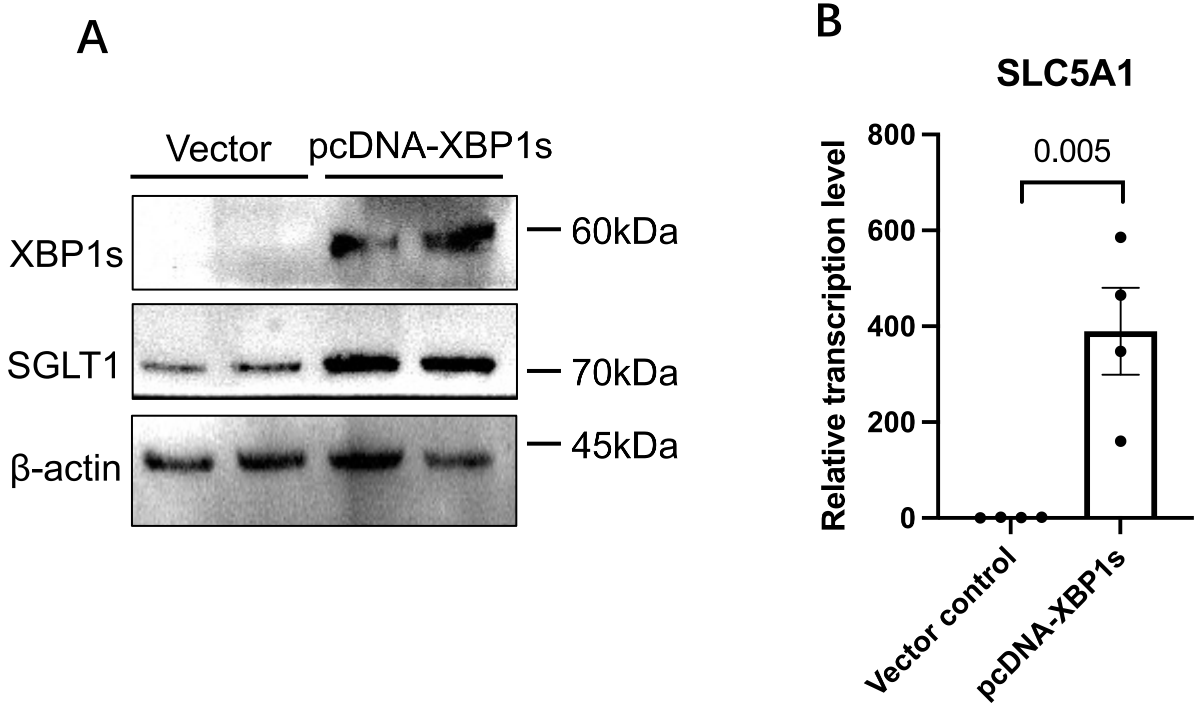

**Supplementary Data**

**Title**: XBP1-mediated transcriptional regulation of SLC5A1 in cystic fibrosis bronchial epithelial cells

**Supplementary Figure 1.** XBP1 upregulates SGLT-1 expression in CFPAC-1-dF cells. (A) CFPAC-1-dF cells were infected with Ad-LacZ or Ad-XBP1s for 48 hours. SGLT-1 and XBP1s protein levels were determined by western blot. (B) CFPAC-1-dF cells were infected with Ad-LacZ or Ad-K907A for 48 hours. SGLT-1, XBP1s, and IRE1α protein levels were determined by western blot.

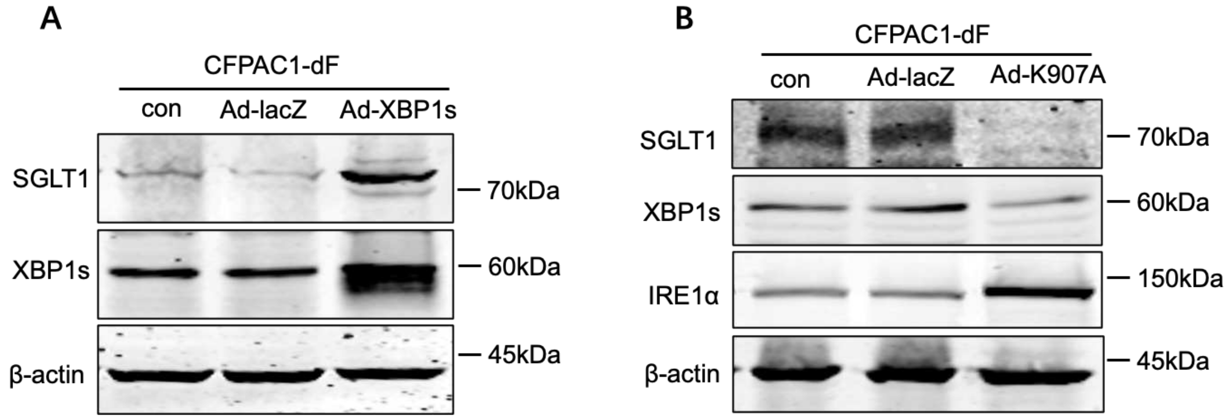

**Supplementary Figure 2.** XBP1 upregulates SGLT-1 expression in HK-2 cells. (A) Western blot of XBP1s, SGLT-1 and β-actin in human kidney 2 (HK-2) cells transfected with the overexpression vector pcDNA-XBP1s or the control empty vector (Vector). (B) Transcription levels of SLC5A1 in HK-2 cells transfected overexpression vector pcDNA-XBP1s or Vector.

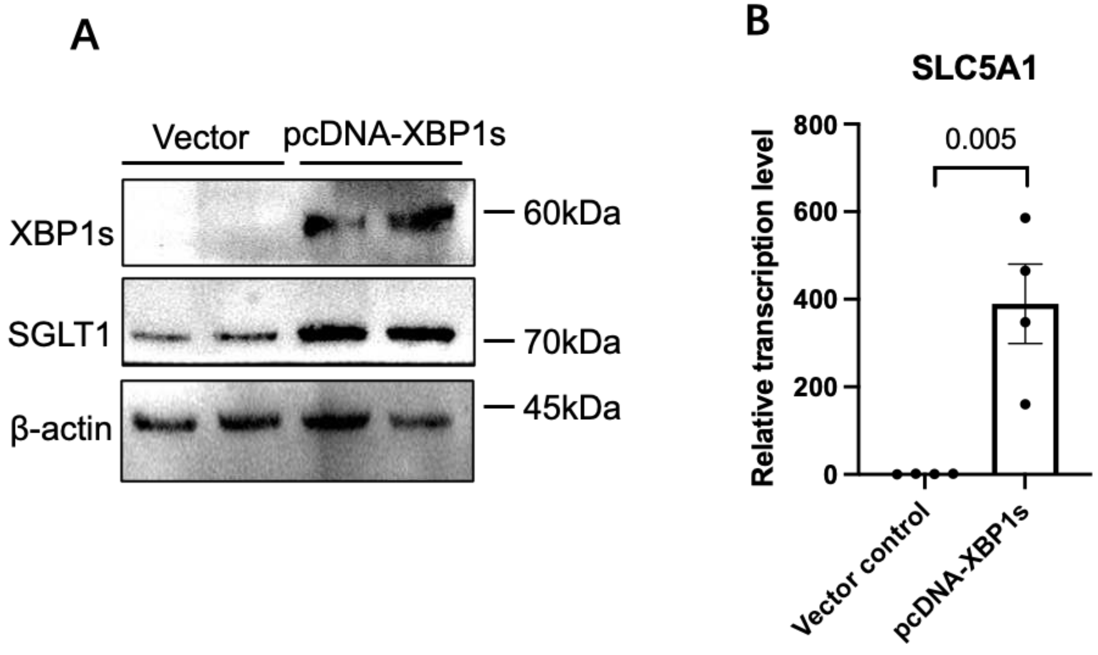

**Supplementary Table 1 (adapted from supplementary ref (1))**

| Drug | IC50 for SGLT-2 (nM) | IC50 for SGLT-1 (nM) | FDA/EMA approval year |
|---|---|---|---|
| Empagliflozin | 3.1 | 8,300 | FDA 2014; EMA 2014 |
| Ertugliflozin | 0.9 | 1,960 | FDA 2017 |
| Dapagliflozin | 1.2 | 1,400 | FDA 2014; EMA 2012 |
| Canagliflozin | 2.7 | 710 | FDA 2013; EMA 2013 |
| Sotagliflozin | 1.8 | 36 | FDA 2023; EMA 2019 |

**Supplemental Table 2: primers used in the present work**

| Gene | Primer sequences for qPCR (h=human) |
|---|---|
| h-SLC5A1 | Forward: TCCTCACCAAACCCATTCCG |
| | Reverse: TCCGCATCCAGGTCAATACG |
| h-GRP78 | Forward: CCTGGGTGGCGGAACCTTCGATGTG |
| | Reverse: CTGGACGGGCTTCATAGTAGACCGG |
| h-IRE1α | Forward: AGAGAAGCAGCAGACTTTGTC |
| | Reverse: GTTTTGGTGTCGTACATGGTGA |
| h-XBP1s | Forward: CCGCAGCAGGTGCAGG |
| | Reverse: GAGTCAATACCGCCAGAATCCA |
| h-GAPDH | Forward: TGAAGGTCGGAGTCAACGG |
| | Reverse: AGAGTTAAAAGCAGCCCTGGTG |
| h-SLC5A1 ChIP | Forward: GTTTCTTCCTCTTTACAGTGGGGG |
| | Reverse: CCAGCACAATCCTAATCTCTTGGC |

**References in the Supplementary Data**

1. Q. Zeng *et al.*, Mechanisms and Perspectives of Sodium-Glucose Co-transporter 2 Inhibitors in Heart Failure. *Front Cardiovasc Med* **8**, 636152 (2021).
